# A Longitudinal Study of Head Circumference Trajectories in Autism and Autistic Traits

**DOI:** 10.1007/s10803-024-06578-x

**Published:** 2024-10-12

**Authors:** Sarah A. Ashley, Kate Merritt, Francesca Solmi, Pedro L. Laguna, Abraham Reichenberg, Anthony S. David

**Affiliations:** 1 Division of Psychiatry, University College London, London, UK; 2 CUBRIC, Cardiff University, Cardiff, UK; 3 Department of Psychiatry, Department of Environmental Medicine and Public Health, MINDICH Child and Health and Development Institute, and Seaver Center for Autism Research and Treatment, Icahn School of Medicine at Mount Sinai, New York, USA

**Keywords:** Autism, ALSPAC, Head Circumference, Neurodevelopmental, Longitudinal

## Abstract

Increased head circumference is an established finding in autism spectrum disorder (ASD); however, it is unclear when this increase occurs, if it persists and whether it manifests across the whole ASD spectrum. Head circumference is a strong predictor of brain size and can therefore provide key insights into brain development in ASD. We used data from the Avon Longitudinal Study of Parents and Children to compare head circumference trajectories from birth to 15 years in children with an ASD diagnosis (*N* = 78, controls = 6,404) or elevated autistic traits as measured using the Social Communication Disorder Checklist (*N* = 639, controls = 6,230). Exploratory analyses were conducted in those with ASD and co-morbid cognitive learning needs (CLN). Children with an ASD diagnosis had larger head circumference from birth across childhood and adolescence compared to controls in univariable (B = 0.69, 95% confidence interval [CI]: 0.28–1.09, *p* = 0.001) and multivariable models (B = 0.38, 95% CI: 0.003–0.75, *p* = 0.048). Differences were more marked in those with co-morbid CLN. Children with elevated autistic traits had significantly smaller head circumference compared to controls. There was weak evidence of group differences when height was included as a covariate. Head circumference trajectories in ASD deviate from control children and persist until adolescence. Autistic traits were associated with smaller head circumference, suggesting distinct growth trajectories between clinical cases from those with non-clinical traits.

**Supplementary Information:**

The online version contains supplementary material available at 10.1007/s10803-024-06578-x.

Autism spectrum disorder (ASD) is a neurodevelopmental disorder characterized by impairments in social interaction and communication and the presence of restricted interests and repetitive behaviours. ASD affects 2–3% of children and adults (Loomes et al., 2017). Head circumference (HC) strongly correlates with brain volume during childhood and into adolescence (Bartholomeusz et al., 2002; Piven et al., 1996). Large HC in children with ASD was reported by Kanner in 1943, and subsequently replicated in several systematic reviews and meta-analyses (Molani-Gol et al., 2023; Redcay & Courchesne, 2005; Sacco et al., 2015). Enlarged HC (> 97th percentile) occurs in a subgroup of individuals with ASD, with the largest effect sizes seen in those with comorbid low IQ (> 70). Differences in HC between ASD and controls is largest in early childhood (10%) and reduces with age (1–2% greater in ASD in adolescence and adulthood). Importantly, this neurobiological abnormality can be detected early in life, before clinical signs and symptoms manifest (Courchesne et al., 2003). However, it remains unclear when altered HC in ASD begins and whether it is time-limited.

Some studies detect increased HC in the first month of life (Fukumoto et al., 2008) while others report increases from 6 months or older (Courchesne et al., 2003; Hazlett et al., 2005; Jane Webb et al., 2007).In contrast, a population-based study found no difference in mean head growth from birth to 12 months in boys with ASD compared to boys without ASD (Surén et al., 2013), whereas another study reports no difference until 36 months, where boys with ASD had larger HC compared to controls (Libero, 2017). The majority of studies are underpowered to assess sex differences, although two studies found smaller HC in girls with ASD compared to girls without ASD between birth and 17 months (Crucitti et al., 2020; Surén et al., 2013) Notably, this study showed that the frequency of extreme head size (1 standard deviation above or below the average) was greater in boys and girls with ASD compared to typically developing children, and that boys with ASD in particular were more likely to have either extremely large or small HC (Crucitti et al., 2020). However, several studies have also noted larger HC in individuals with ASD regardless of sex between the ages of 2–16 years (Fombonne et al., 1999; Sacco et al., 2007). These studies highlight the large variability present across studies, and the possibility of subgroups driving these physiological differences. (Constantino et al., 2010; Surén et al., 2013).

There is limited research examining HC trajectories in older children and adolescents with ASD. A cross-sectional study reports larger HC in both children and adults with ASD (8–46 years) (Aylward et al., 2002), consistent with findings of larger HC in adult males with ASD (20 + years) (Denier et al., 2022). Using total brain volume (TBV) as a proxy measure for HC, boys with ASD and disproportionately larger TBV to height (i.e., > 1.5 standard deviations) at 3 years, displayed persistently larger TBV at older ages (4, 5 years, and 11 years) compared to controls (Lee et al., 2021). However, there were no differences between girls with and without ASD in TBV at any timepoint (Lee et al., 2021).

It is unclear whether abnormal HC represents a brain-specific process, or more generalized overgrowth (Chawarska et al., 2011; Dawson et al., 2007). In infancy (< 2 years), one review reports that both HC and height are enlarged in infants with ASD compared to controls (Molani-Gol et al., 2023). Studies across broader age ranges also report that height is a strong predictor of HC in ASD and should be included as a covariate (Chaste et al., 2013; Chawarska et al., 2011; Mraz et al., 2007; Sacco et al., 2007). In contrast, some studies suggest that HC is enlarged relative to height (Lainhart et al., 2006; Miles et al., 2000), with one study spanning a large age range (3–47 years) citing no effect of age, sex or non-verbal IQ on HC in ASD (Lainhart et al., 2006).

Previous studies have had several limitations: first, the majority are based on clinical ASD samples, which may not be representative, and obtained HC data retrospectively. Second, most studies investigated HC cross-sectionally or across limited timepoints. Third, some studies did not consider potentially important confounders which may relate to the variability of findings.

To address these limitations, we used general population data from the Avon Longitudinal Study of Parents and Children (ALSPAC) to investigate differences in HC from birth through to 15 years in children with ASD and controls, adjusting for important confounders. Analyses were repeated in children with elevated autistic traits to investigate HC across the full ASD spectrum.

## Methods

### Sample

ALSPAC recruited pregnant women resident in Avon, UK, with delivery dates between April 1991-December 1992 (Boyd et al., 2013; Fraser et al., 2013). The total cohort comprised 15,645 children excluding triplets and quadruplets for anonymity (Northstone et al., 2019; Solmi et al., 2020).

This study included children who had at least one HC measurement, as well as complete data on diagnosis (ASD or autistic traits) and confounders.

The study website contains details of all data: http://www.bristol.ac.uk/alspac/researchers/our-data/. Ethical approval was granted by the ALSPAC Ethics and Law Committee (ALEC) and the Local Research Ethics Committees. Informed consent for the use of data collected via questionnaires and clinics was obtained from participants following the recommendations of the ALEC.

### Outcome

The primary outcome measure was HC trajectories in individuals with autism, elevated autistic traits, and controls, and to investigate whether diagnosis status explains changes in HC. HC measurements were recorded for the whole ALSPAC cohort at birth to 1 year; age 7 and 15 years. Additional HC measures between 4 and 61 months were available in a 10% subset of the original sample. Analyses only included timepoints in which HC measurements were available for at least 10 participants per group (ASD/autistic traits/controls). From an initial 100 timepoints, 19 timepoints met this criterion which were used in all analyses except for those involving ASD and CLN due to small sample sizes. From birth to 12 months, HC and height were available at the following age in months for individuals with ASD: birth, 1–4, 8, 9, 12. All months were available from birth-12 months in the autistic trait analyses. HC data were collected by researchers supplemented by routine measurements by nationally mandated local health services. If multiple HC measures were available for a specific month, the average value was calculated. Values were excluded if measurements differed by > 3 cm within a month. Outliers were identified using cut-offs from the WHO Child Growth Standards (De Onis, 2006; Shi et al., 2018).

### Diagnosis

#### ASD Diagnosis

Participants with ASD were identified through parent-reports of whether their child had ever received a diagnosis of ASD, when the child was 9 years-old. Those who replied ‘no’ were classed as controls. This measure has strong sensitivity (95%) and specificity (99%) in identifying clinical ASD (Hall et al., 2023; Russell et al., 2015).

Main analyses used the case-control definition above (ASD vs. controls). However, we also investigated the influence of ASD comorbid for cognitive learning needs (CLN), defined as individuals with moderate, severe and profound learning difficulties, as well specific difficulties such as dyslexia or dyspraxia. Records were retrieved from the National Pupil Leave Annual School Census for 2003/4 (Department for Education and Skills, 2005). In accordance with ALSPAC confidentiality guidelines, these data are aggregated across all cognitive levels (Golding et al., 2001). Of the analytic sample (*n* = 6,482), 76.4% of participants (*n* = 4,952) also had data available on CLN. Those without CLN data either did not attend a state school in England, could not be matched (e.g., changes in personal information), or there were legal restrictions which prevented linkage (Madley-Dowd et al., 2022).

#### Autistic Traits

The Social and Communication Disorders Checklist (SCDC) used parental reports when children were 7.5 years (Skuse et al., 2005). The SCDC is a 12-item questionnaire which measures social reciprocity and social communication difficulties. In line with previous research, children scoring > 7 were defined as having elevated autistic traits. The SCDC has excellent internal consistency (α = 0.93) and high test-retest reliability (*r* = 8.1, mean interval 2.7 years), as well as good sensitivity and specificity for ASD (Skuse et al., 2005).

### Confounders

We selected confounders based on previous literature showing associations with both HC and ASD (Chaste et al., 2013; Modabbernia et al., 2017; Zwaigenbaum et al., 2014). These included: age, sex, maternal body mass index (BMI), gestational age, weight-at-birth and maternal highest-level-of-education. To control for general growth, we added length/height as a time-varying covariate (available at all timepoints?).

### Data Analysis

All analyses were conducted in Stata17 (StataCorp, 2017).

We applied univariable and multivariable multilevel linear mixed regressions to model repeated time observations within participants, to compare HC trajectories in children with/without an ASD diagnosis and elevated autistic traits. We included a random intercept on child and a random slope on linear age. First, we fitted two unconditional models progressively including a mean-centred indicator of child age in months (unconditional model 1) and age^2^ (unconditional model 2) to assess the trajectory of HC over time. Model fit was assessed using Akaike Information Criterion (AIC) and Bayesian Information Criterion (BIC). If there was evidence of a non-linear association with age (i.e., *p* < 0.05) along with improved model fit (i.e., lower AIC and BIC values), we retained both the linear and squared-age indicators in the final model. Output for unconditional models is listed in the supplement.

Next, we fitted a univariable model which included ASD diagnosis or elevated autistic traits; multivariable (model 1) as well as the two age indicators. Subsequently, we fitted a series of multivariable models progressively adjusting for participant’s sex (model 2); maternal education (model 3), maternal BMI (model 4), gestational age and birth weight (model 5). We also examined the following interactions with group in three separate models: (i) age, (ii) age and age-squared, and (iii) sex.

For comparisons between the analytic samples and full ALSPAC cohort in terms of demographics and confounders, see supplemental materials (Table S1).

### Secondary Analyses

We included height (model 6) to assess whether altered HC may reflect generalized growth as opposed to head/brain specific growth.

Due to limited statistical power, we selected univariable model 3 to examine mean trajectory differences between ASD with/without CLN compared to controls (ASD with CLN *n* = 13; no ASD *n* = 4,906; total analytic sample *N* = 4,919). Additionally, we investigated the impact of removing those with CLN from the main ASD analyses and explored within-group differences by comparing ASD with CLN vs. ASD without CLN.

To assess the time when group differences in HC emerge, we fitted post-hoc unadjusted and FDR adjusted two-sample t-tests to timepoints between the ages of birth-12 months, and also at age 15.5 years to see if differences persist (see supplement). To examine general growth effects, t-tests of height differences at these ages were also performed.

### Sensitivity Analyses

To improve comparability between groups, we restricted analyses to participants who had complete data for both ASD and autistic traits. Finally, to avoid biasing the results from the autistic traits group with individuals with ASD, we removed those with ASD from the autistic traits group (i.e., elevated autistic trait group consisted of individuals above the cut-off for the SCDC but no ASD diagnosis) and compared those with controls below the SCDC cut-off.

## Results

### Sample

Of the 15,645 children within ALSPAC (*n* = 14,442 excluding twins), 7,695 had data on ASD, with 95 children reported to have a clinical diagnosis of ASD. All 95 children had at least one HC measure, and 78 also had complete data for confounding variables (controls = 6,404). Most ASD cases were male (78%), whereas controls were evenly split by sex (50% male). ASD and controls were comparable across demographic characteristics and confounders, although mean height at 15.5 years was higher in the ASD group (Table 1). Per participant, the mean number of timepoints with HC data was 4, with a median of 3 (SD = 3.22; range = 1–18, interquartile range = 3). Compared to the full ALSPAC cohort, the demographic characteristics for the two analytic samples (ASD and autistic traits) shared comparable distributions. However, mean gestational age and birth weight were slightly lower in the full cohort compared to the analytic sample (Table S1). For sampling numbers at each HC measurement timepoint, see tables S2 and S3.

**Table 1:** Characteristics of the analytical sample, overall and by case/control status. Sample based on participants with ASD or autistic traits and complete confounder data

	ASD analytic sample	ASD absent	ASD present	Autistic traits analytic sample	Autistic traits absent	Autistic traits present
	*N*(%)	*n*(%)	*n*(%)	*n*(%)	*n*(%)	*n*(%)
Total	6,482 (100%)	6,404 (98.80%)	78 (1.20%)	6,869	6,230 (90.70%)	639 (9.30%)
**Sex**						
*Male*	3,239 (49.99%)	3,178 (98.12%)	61 (1.88%)	3,519	3,111 (88.41%)	408 (11.59%)
*Female*	3,243 (50.01%)	3,226 (99.48%)	17 (0.52%)	3,350	3,119 (93.10%)	231 (6.90%)
**Ethnicity**						
*White*	6,139 (94.72%)	6,062 (98.75)	77 (1.25)	6,507	5,900 (90.67%)	607 (9.33%)
*Ethnic minority*	228 (3.52%)	228 (100%)	0	240	219 (91.25%)	21 (8.75%)
*Missing*	115 (1.76%)					
**Maternal highest educational attainment**						
*Compulsory*	3,710	3,668 (98.87%)	42 (1.13%)	3,948	3,546 (89.82%)	402 (10.18%)
*Non-compulsory*	2,772	2,736 (98.70%)	36 (1.30%)	2,921	2,684 (91.89%)	237 (8.11%)
	**Mean (SD)**	**Mean (SD)**	**Mean (SD)**	**Mean (SD)**	**Mean (SD)**	**Mean (SD)**
Gestational age (in weeks)	39.52 (1.73)	39.52 ()	39.49 (2.36)	39.51 (1.76)	39.51 (1.75)	39.47 (1.88)
Birth weight (grams)	3478.10 (518.25)	3446.02 (517.07)	3478.10 (608.52)	3438.76 (526.01)	3441.65 (523.48)	3410.76 (549.75)
Length at birth (cm)	50.89 (2.38)	50.89 (2.38)	51.03 (2.55)	50.88 (2.39)	50.90 (2.37)	50.70 (2.54)
Height at 15.5 years (cm)	169.37 (7.85)	169.34 (8.24)	172.91 (7.85)	169.40 (8.38)	169.37 (8.33)	169.69 (8.94)
Age in years at ASD/SCDC measurement	9.65 (1.52)	9.64 (1.23)	9.65 (1.52)	91.88 (1.69)	91.84 (1.64)	91.89 (1.58)
Maternal pre-pregnancy BMI	22.88 (3.68)	22.88 (3.68)	22.65 (3.67)	22.86 (3.67)	22.86 (3.66)	22.86 (3.77)
Average number of head circumference measurements	5 (3.25)	5 (3.24)	5(3.75)	6 (4.40)	6 (4.42)	6(4.14)

Note: BMI, body mass index; SCDC, Social and Communication Disorders Checklist; We collapsed maternal highest educational attainment into a binary variable due to small sample size and avoid possibility of participant identification. Analyses used the non-collapsed version consisting of 5 levels of educational attainment

There were 7,813 children with outcome data on autistic traits using the SCDC, 759 of which were classified as having elevated traits. All but two had at least one HC measurement, and 639 had complete data for confounders (controls = 6,230). Most children with elevated autistic traits were male (64%); controls were evenly split by sex (50%). Within participants, the mean number of HC timepoints was 5, with a median of 4 (SD = 4.40; range = 1–27, interquartile range = 5). There were no significant differences in demographic characteristics for those with elevated autistic traits compared to controls (Table 1).

### Trajectories of Head Circumference (HC) by ASD Diagnosis

**Fig. 1:**
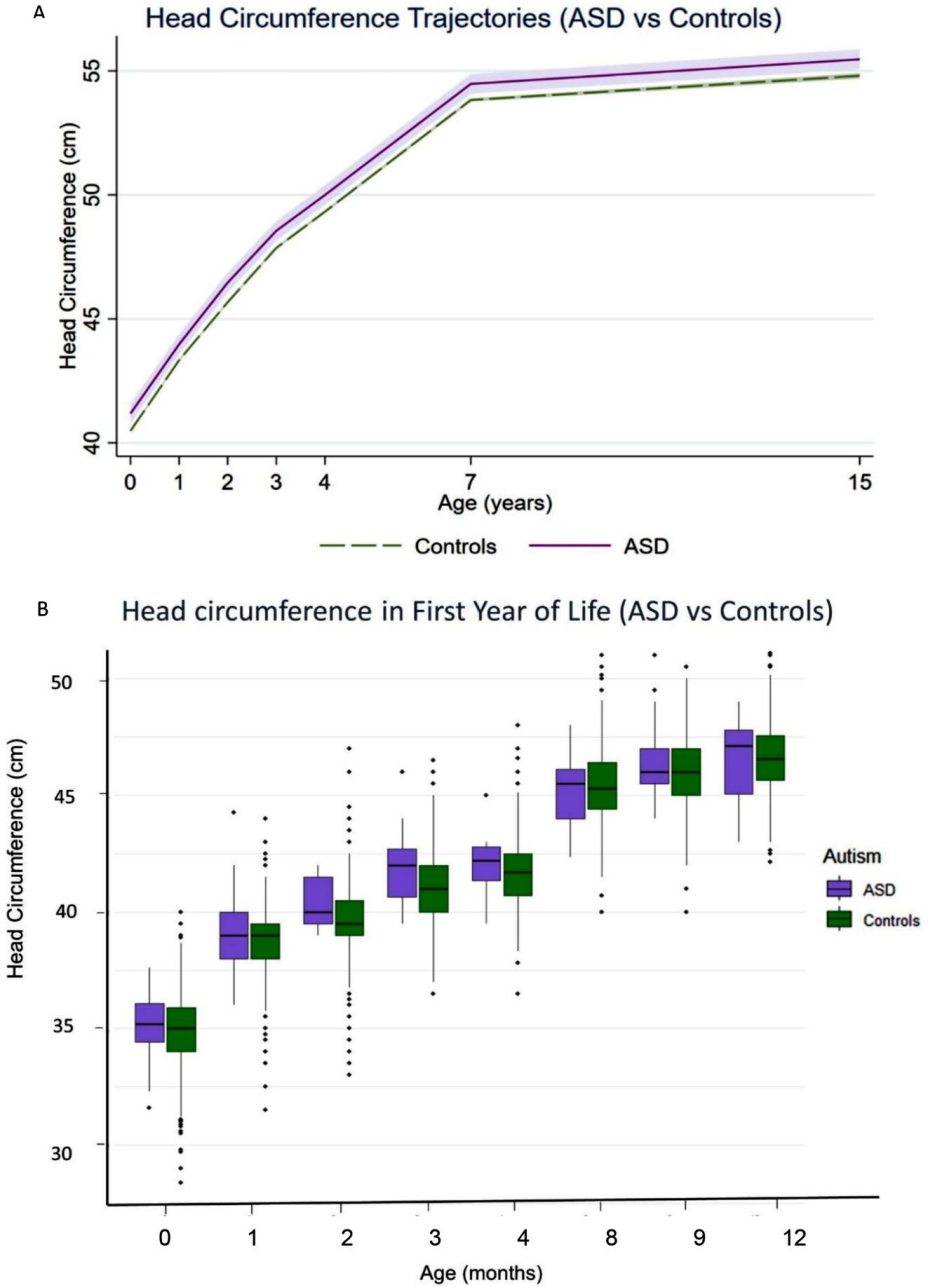
Head circumference (HC) trajectories (cm) in ASD cases (purple) and controls (blue). Plot A shows modelled mean HC values (model 2, adjusted for sex) with 95% confidence intervals shown in lilac for ASD and light green for controls. (*N* = 6,482). Plot B is boxplot of HC measurements from birth to 12 months in ASD (purple) and controls (green). For clarity, integer numbers have been used in both plots to represent age in which HC measurements were collected

In the univariable model, participants with ASD had larger HC compared with controls (B = 0.69, 95% confidence interval [CI]: 0.28–1.09, *p* = 0.001; Fig. 1; Table 2). After adjusting for sex, there was still evidence of an association between ASD and larger HC trajectory, although the magnitude of the association was reduced (B = 0.41, 95% CI: 0.02–0.80, *p* = 0.038). Results remained largely unchanged after adjustment for maternal education, maternal BMI, gestational age, and birth weight. There was no evidence for effect modification by sex, age or age-squared.

**Table 2:** Head circumference (HC) trajectories in ASD vs. controls showing increase in HC in ASD. Univariable and multivariable linear mixed regressions assessing HC in ASD versus controls. Sample based on timepoints in which at least 10 people per group (ASD or controls) were present at each timepoint and complete data on the ASD measure. (Analytic sample *N* = 6,482, ASD = 78, controls = 6,404)

	Head circumference trajectories in ASD vs. controls		
	Beta Coefficient	95% Confidence intervals	*P*
Univariable model 1	0.69	0.28–1.09	0.001
Model 2: model 1 + child’s sex	0.41	0.02–0.80	0.038
Model 3: model 2 + maternal education	0.41	0.02–0.80	0.039
Model 4: model 3 + maternal BMI	0.42	0.03–0.81	0.034
Model 5: model 4 + gestational age and birth weight	0.38	0.003–0.75	0.048
Model 6: model 5 + height	0.06	-0.21–0.33	0.66

Unconditional Model 1 (age) and Model 2 (mean centred age and age squared) are presented in supplemental material. For univariable model 1 onwards, we incorporated random effects of participant id and mean centred age. Univariable model 1 consisted of ASD and time variables. Interactions between ASD and age (*p* = 0.87), age squared (*p* = 0.58), and sex (*p* = 0.42) were non-significant and therefore the group coefficient was not included in this table

#### Secondary Analyses

After adjusting for height, there was no longer evidence of a difference in HC between ASD cases and controls (B = 0.06, 95% CI: -0.21–0.33, *p* = 0.66).

When investigating differences in trajectories of HC in children with comorbid ASD and CLN compared to controls (supplementary figure S1), there was evidence of larger HC in the ASD CLN group (univariable model 3: B = 1.69, 95% CI: 0.75–2.63, *p* < 0.0001). This coefficient was approximately four times larger than when controls were compared to ASD without CLN (B = 0.45, CI: 0.03–0.87, *p* = 0.036). Finally, when investigating the differences in HC trajectories between the two ASD groups, there was evidence of differences between the groups, with larger HC in the ASD with CLN group compared to ASD without CLN (B = 1.70, 95% CI: 0.76–2.64, *p* < 0.0001).

When examining individual timepoints, group differences in HC were observed from 2 months (t[1772]=-2.69, *p* = 0.007), differences in height occurred later (9 months; t[3115]=-2.43, *p* = 0.015; see supplementary Table S4). Analyses did not survive corrections for multiple comparisons.

#### Sensitivity Analyses

Results were similar to the main analyses when participants had complete data for both diagnoses (ASD and autistic traits), i.e., there was evidence of larger HC in autism compared to controls. See supplement Table S5.

### Trajectories of Head Circumference (HC) in Elevated Autistic Traits

**Fig. 2:**
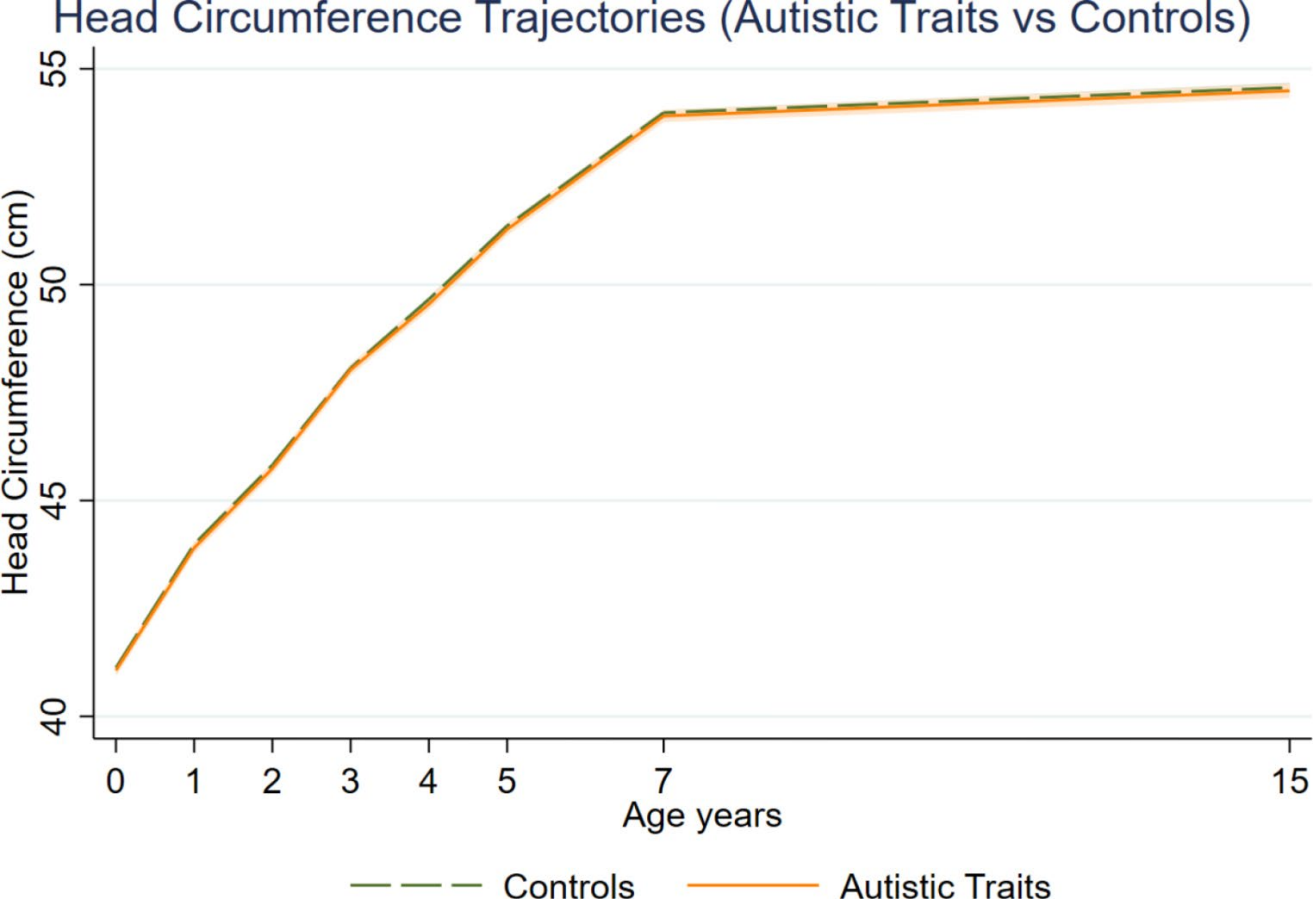
Head circumference (HC) trajectories in participants scoring high on the autistic traits measure (SCDC score of 8 or above) in comparison to controls. Trajectories show modelled mean values when adjusting for sex (Model 2) with 95% confidence intervals shown in orange for the autistic traits group and green for controls. Note that confidence intervals overlap between autistic traits group and controls. (*N* = 6,869)

In univariable analyses there was no evidence that mean HC differed between the autistic traits group and controls (B=-0.08, 95% CI: -0.22–0.06, *p* = 0.28, Table 3; Fig. 2). After adjusting for sex, the magnitude of the difference increased and there was weak evidence that participants with higher autistic traits had *lower* HC values compared to controls (B=-0.22, 95% CI: -0.36–-0.09, *p* = 0.001). Results remained unchanged after adjusting for maternal education and maternal BMI, but the magnitude of effect was reduced after gestational age and birthweight were included in the model (model 5: B =-0.16, CI: -0.28–0.03, *p* = 0.016). There was no evidence for interactions between autistic traits group and age, age-squared and sex.

**Table 3:** Head circumference (HC) trajectories in autistic traits vs. controls showing decrease in HC in autistic traits. Univariable and multivariable linear mixed regressions assessing HC in autistic traits versus controls. Sample based on timepoints with at least 10 participants per group, and those with complete data on the autistic traits measure. (Analytic sample *N* = 6,869, autistic traits = 639, controls = 6,230)

	Head circumference trajectories in Autistic Traits vs. Controls		
	Beta Coefficient	95% Confidence intervals	*P*
Univariable model 1	-0.08	-0.22–0.06	0.28
Model 2: model 1 + child’s sex	-0.22	-0.36 - -0.09	0.001
Model 3: model 2 + maternal education	-0.20	-0.33 - -0.08	0.003
Model 4: model 3 + maternal BMI	-0.20	-0.33 - -0.07	0.003
Model 5: model 4 + gestational age and birth weight	-0.16	-0.28 - -0.03	0.016
Model 6: model 5 + height	-0.10	-0.20–0.002	0.055

Unconditional Model 1 (age) and Model 2 (mean centred age and age squared) are presented in supplemental material. For univariable model 1 onwards, we incorporated random effects of participant id and mean centred age. Univariable model 1 consisted of ASD and time variables. Interactions between ASD and age (*p* = 0.54), age squared (*p* = 0.39), and sex (*p* = 0.20) were non-significant and therefore the group coefficient was not included in this table

#### Secondary Analyses

When adjusting for height, the model showed weak evidence for an association (height model 6: MD = -0.10, CI: 0.20–0.001, *p* = 0.055). When examining individual timepoints, smaller HC in the elevated autistic traits group were observed at 6 months (t[269] = 2.01, *p* = 0.045) and 7 months (t[319] = 3.23, *p* = 0.001), differences in height occurred at 3 months (t[942] = 2.15, *p* = 0.032; see Table S6). Analyses did not survive corrections for multiple comparisons, apart from HC at 7 months (*p* = 0.014).

#### Sensitivity Analyses

Results were similar to the main analyses when participants had complete data for both diagnoses (ASD and autistic traits), i.e., there was evidence of smaller HC in those with elevated autistic traits compared to controls. See supplement Table S7. Likewise, when individuals with ASD were removed from trait analyses, smaller HC was observed in those with elevated traits (Table S8).

## Discussion

We examined HC trajectories between birth and 15-years in young people with ASD and those with elevated autistic traits compared to controls. Children with ASD had significantly larger HC across this period. Differences in HC were attributed to generalized dysregulation of growth in those with ASD. Conversely, children with elevated autistic traits had reduced HC compared to controls. These preliminary findings go against the idea of a pathophysiological continuum of autistic traits and ASD, highlighting potentially distinct aetiological differences that may separate clinical versus non-clinical cases.

Previous research on HC in ASD populations is typically restricted to the first 2–3 years of life (Constantino et al., 2010), and few studies prospectively chart HC beyond this period. Our study provides evidence that HC is consistently larger in ASD, as demonstrated by the lack of an age-by-group interaction, whilst also being robust against adjustment for multiple confounders. The timing of this increase has remained elusive in the literature, with some studies reporting the onset between 1 and 3 months-of-age consistent with the findings from our study (Fukumoto et al., 2008; McKeague et al., 2015), whereas others report enlarged HC from 6 months onwards (Courchesne et al., 2003; Hazlett et al., 2005; Webb et al., 2007). Our study supports the view that these changes in HC are continuous and not time-limited to early development.

Previous literature has suggested that enlarged HC may only affect a subgroup of individuals with ASD. For example, one meta-analysis reported macrocephaly (HC > 97th percentile) in 15.7% of individuals with ASD (versus 3% controls) and was more common in individuals with IQ scores < 70 (Sacco et al., 2015). In our subgroup analyses, the largest effect-size differences between ASD and controls were observed when participants with ASD had CLN, suggesting that shared mechanisms might lead to larger HC and compromised intellectual functioning, or alternatively that cognitive impairments may be an important mediator in larger HC within ASD. Importantly, enlarged HC was observed in the ASD group regardless of additional CLN, however the coefficient size was reduced when the CLN group were removed. In the general population, head growth during infancy is a significant predictor of later IQ (Gale et al., 2006). A birth cohort study found greater increases in HC and height during the first 5 years of life were associated with higher IQ in childhood, whereas greater weight gain after 1 year was not (Kirkegaard et al., 2020). Our study suggests there may be an opposing effect of IQ on head growth in ASD samples, however confirmation will require larger samples.

Whether large HC in ASD is related to a more general marker of dysregulated growth is debated. When controlling for height, there were no longer group differences between ASD and controls in HC, suggesting that general growth, irather than brain growth specifically, may be dysregulated in ASD. This is consistent with a review (Green et al., 2015), citing increased physical growth - including HC, height, weight and BMI - in children with ASD compared to typically developing individuals. The review also highlights that due to the paucity of longitudinal studies in adolescent and adulthood, it is unknown whether height and weight follow relative increased acceleration in childhood followed by deceleration in those with ASD as seen for HC and brain growth. The timing of dysregulation may differ for height compared to HC, as increases to HC according to unadjusted post-hoc t-tests occurred earlier (at 2 months) than height increases (at 9 months) in those with ASD. These results were no longer significant when controlling for multiple comparisons, however it is also possible that small sample sizes at individual timepoints lack sufficient statistical power. This contrasts to one study which reported overgrowth in length/height earlier at 4-months, and increased HC at 8-months in ASD (Campbell et al., 2014). Some studies report that HC in ASD varies independently of height (Dawson et al., 2007; Miles et al., 2000), while others suggest that they covary, reflecting more generalised growth processes (Chaste et al., 2013; Chawarska et al., 2011; Dissanayake et al., 2006). Our findings suggest that general growth, including HC trajectories, are altered in ASD.

There are several possible reasons for the association between autistic traits and *reduced* HC. First, the aetiology of ASD may not be best explained through a continuum but instead by distinct symptom profiles. Whist research has shown that the SCDC has strong specificity and sensitivity to detect ASD, only 65% of the autistic traits group were diagnosed with ASD in our sample. The SCDC predominately captures deficits in social communication/reciprocity and does not include items on repetitive behaviours or restricted interests. There is evidence linking enlarged brain size during childhood in ASD and poor performance on repetitive behaviour scales, as well as with delayed language and skill regression (Courchesne et al., 2003; Lainhart et al., 2006; Nordahl et al., 2011). It is therefore possible that neurobiological mechanisms underlying enlarged HC show a greater affinity for certain clinical outcomes over others. Furthermore, it is possible that the SCDC captures general psychopathology, as social difficulties are common across many disorders. A more detailed clinical assessment of the SCDC group is needed to ascertain whether the reductions in HC can be attributed to autistic traits or other disorders.

HC and brain size are strongly correlated (Bartholomeusz et al., 2002; Hshieh et al., 2016; Piven et al., 1996), hence HC can be used as a proxy measure of neurodevelopment.^37^ Atypical cell proliferation and reductions in neuronal pruning are thought to underlie the growth differences in HC observed in ASD and cause an overabundance of cortical neurons, particularly in frontal and temporal brain networks which underlie social and language processes (Courchesne et al., 2018). The are several potential genes associated with ASD which affect brain size and growth in general (e.g., mutations to chromodomain helicase DNA binding protein 8 (CHD8) (Sugathan et al., 2014) (Adam et al., 2022) However, while larger HC may be a viable biomarker for ASD, it is also a biomarker for several other genetic conditions (McCaffery & Deutsch, 2005).

Several limitations should be considered when interpreting this study. As with most longitudinal cohorts, missing data reduced sample size and representativeness. The majority of participants (94%) were of white ethnicity, our findings therefore have limited generalizability to other ethnic groups. The use of parent confirmation of a diagnosis of ASD may be less valid than formal diagnostic tools. However, the number of ASD cases was similar to that of previous ALSPAC studies which used multidisciplinary assessments from health and educational records (Williams et al., 2008). The subgroup of ASD with CLN was small and may lead to the model overfitting the data when regression models are used, and so these findings require replication in a larger sample. Greater clinical profiling of the ASD and autistic traits group beyond CLN subgroups would be useful. We did not observe any sex-by-group interactions with HC, although we were likely underpowered to do so due to females representing only 21% (*n* = 15) of the ASD sample. Strengths of the study include the use of a representative birth cohort with multiple (> 10) timepoints for HC measurements. Previous longitudinal studies typically examine 3–4 timepoints, often limited to early development (Dissanayake et al., 2006; Fukumoto et al., 2008; Hazlett et al., 2005).

Findings from this study are consistent with previous literature that large HC may be considered an accessible biomarker for ASD, at least for a subgroup of individuals with ASD, and potentially complement behaviour assessments for diagnosis throughout childhood (Denier et al., 2022; Muratori et al., 2012; Sacco et al., 2015). Greater profiling of ASD growth and clinical trajectories is required to gain a better picture of this subgroup, particularly in adulthood. Our findings suggest that HC enlargement is specific to those with clinical diagnoses as we do not find evidence of enlargement in the subclinical trait sample. It will be important for future studies to continue to monitor HC across a greater time-period to see the full extent of the developmental trajectory.

## Electronic supplementary material

Below is the link to the electronic supplementary material.

Supplementary Material 1

Supplementary Material 2
