# Supplementary material for: A Longitudinal Study of Head Circumference Trajectories in Autism and Autistic Traits
Source: J Autism Dev Disord. 2024 Oct 12;56(2):808–18. doi: 10.1007/s10803-024-06578-x (PMC12864280; doi:10.1007/s10803-024-06578-x)
Supplement: Supplementary file 1 — Supplementary Material 1 [file 10803_2024_6578_MOESM1_ESM.docx]

# Supplementary Materials

## Unconditional models

In our unconditional models using the ASD/Control sample (Table 2 of main manuscript), there was evidence of a non-linear association with age (age^2^ p < 0.0001) and improved model fit when incorporating linear and squared age indicators (AIC = 190958.5, BIC = 190984) compared to solely using linear age (AIC = 210966.8, BIC = 211017.9). Consequently, we retained linear and non-linear age in univariable and multivariable models. AIC and BIC values remained largely unchanged beyond this point across models.

In our unconditional models using the Autistic traits/Controls sample (Table 3 of main manuscript), there was evidence of a non-linear association with age (age^2^ p < 0.0001) and improved model fit when incorporating linear and squared age indicators (AIC = 244545.5, BIC = 244606.9) compared to solely using linear age (AIC = 268695, BIC = 268747.6). Consequently, we retained linear and non-linear age in univariable and multivariable models. AIC and BIC values remained largely unchanged beyond this point across models.

## Missing data and outliers

Among 15,645 participants, a total of 6,482 participants (41.49%) had complete data for autism and covariates, and 6,869 participants (43.92%) had complete data on autistic traits and covariates (see figures S2, S3 and S4 for sample flowcharts). Individuals with missing data on autism diagnosis had younger gestational age (M = 37.27 weeks vs M = 39.52) and mothers that were less likely to engage in higher education (i.e., 1,334 vs 2,772) compared to the main analytic sample. Pre-pregnancy BMI was similar across groups, although marginally larger in those with missing ASD data (M = 23.00 vs M = 22.88). Compared to the full ALSPAC sample (**Table S1**), the analytic samples used in the ASD and autistic trait analyses shared similar demographic characteristics. Small differences between groups were identified for gestational age and birth weight, both of which were lower in the full ALSPAC sample compared to the analytic samples.

Based on individuals with complete data on both clinical outcomes and covariates, there were 45 observations that were outliers: head circumference (32 observations), height (6 observations) or birth weight (7 observations) values. This resulted in the removal of 2 participants from ASD analyses and 1 in autistic traits analyses (2 when participants had complete data on clinical outcomes). Additionally, we removed observations in which differences in head circumference or height if collected at the same timepoint were over 3 cm, this resulted in the removal of 45 observations for head circumference and 104 timepoints for height.

**Table S1. Characteristics of the ASD and autistic traits analytic samples compared to the full ALSPAC cohort (excluding non-singleton births n = 203).**

|  | Full ALSPAC cohort | Analytic sample (ASD analyses) | Analytic sample (autistic trait analyses) |
| --- | --- | --- | --- |
|  | N (%) | n (%) | n (%) |
| Total | 15,442 (100%) | 6,482 (100%) | 6,869 (100%) |
| **Sex** |  |  |  |
| *Male* | 7,583 (49.10%) | 3,239 (49.99%) | 3,519 (51.25%) |
| *Female* | 7,262 (47.02%) | 3,243 (50.01%) | 3,350 (48.75%) |
| *Missing* | 597 (3.87%) |  |  |
| **Ethnicity** |  |  |  |
| *White* | 11,379 (73.62%) | 6,139 (94.80%) | 6,507 (94.73%) |
| *Ethnic minority* | 604 (3.91%) | 228 (3.52%) | 240 (3.49%) |
| *Missing* | 3,459 (22.46%) | 343 (5.28%) | 122 (1.78%) |
| **Maternal highest education** |  |  |  |
| *Compulsory/Vocational/None* | 7,975 (51.62%) | 3,710 (57.29%) | 3,948 (57.54%) |
| *Non-compulsory* | 4,350 (28.12%) | 2,772 (42.71%) | 2,921 (42.46%) |
| *Missing* | 3,117 (20.26%) |  |  |
|  | Mean (SD) | Mean (SD) | Mean (SD) |
| Gestational age (weeks) | 38.41 (5.49) | 39.52 (1.73) | 39.51 (1.76) |
| Birth weight | 3393.01 (570.72) | 3478.10 (518.25) | 3438.76 (526.01) |
| Length at birth | 50.80 (2.69) | 50.89 (2.38) | 50.88 (2.39) |
| Height at age 15.5 years | 169.23 (8.36) | 169.37 (7.85) | 169.40 (8.38) |
| Maternal pre-pregnancy BMI | 22.93 (3.84) | 22.88 (3.68) | 22.86 (3.67) |

**Table S2**. Sample size for ASD versus Controls at head circumference (HC) measurement timepoints between birth and 15.5 years.


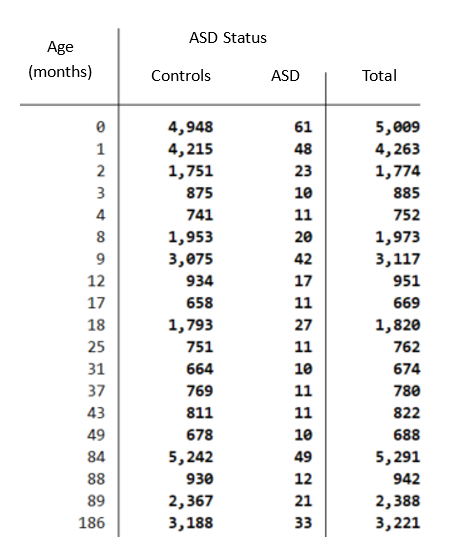


**Table S3**. Sample size for elevated autistic traits group versus Controls at head circumference (HC) measurement timepoints between birth and 15.5 years.


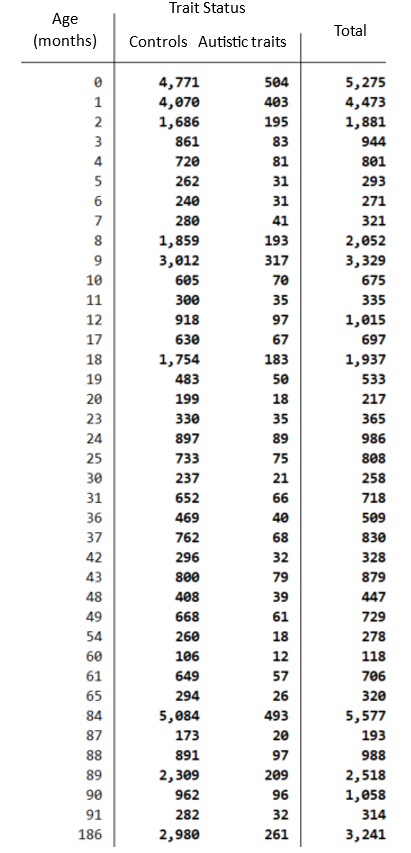


### ASD with Cognitive Learning Needs

When investigating differences in trajectories of head circumference in children with comorbid ASD and CLN compared to controls (supplementary figure S1), there was evidence of larger HC in the ASD CLN group (univariable model 3: B=1.69, 95% CI: 0.75–2.63, p<0.0001).


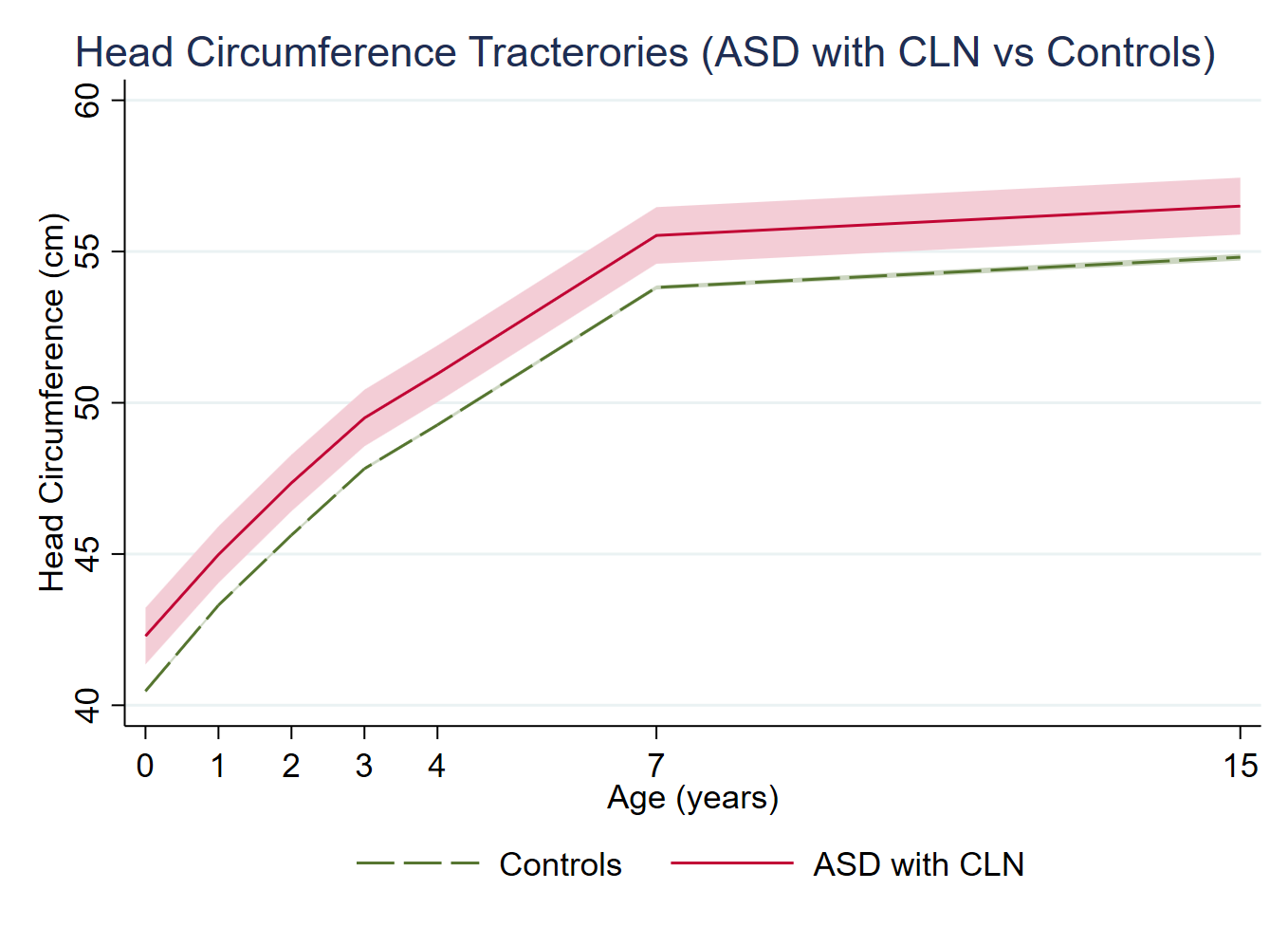
Figure S1. Head circumference trajectories in participants with ASD and CLN compared to controls (no ASD). Trajectories show modelled mean values when adjusting for sex (Model 2) with 95% confidence intervals in pink for ASD + CLN and green for controls. (N = 6,417)

### Head circumference and height across first 12 months in ASD group: T-tests

When examining individual timepoints, head circumference did not significantly differ between ASD and controls at birth (t[5007] = -1.48, p = 0.14) and 1 month (t[4261] = -1.75, p = 0.08; **Table S4**), but significantly differed at 2 months (t[1772] = -2.69, p = 0.007), 3 months (t[883] = -1.97, p = 0.049), and 9 months (t[3115] = -2.35, p = 0.02) with larger head circumference in ASD compared to controls by approximately 1cm. Head circumference remained larger at age 15 years in those with ASD (t = -2.02, p = 0.043). There was no evidence of group differences in length/height from birth to 8 months (p > 0.05), however mean height at 9 months (t[3115] = -2.43, p = 0.015) and 15 years (t[3219] = -2.47, p = 0.013) was higher in ASD compared to controls.

| **Table S4.** T-tests of head circumference values from birth to 12 months in individuals with ASD and controls. Head circumference at age 15.5 years is also reported. | | Head Circumference in ASD vs Controls | | |  | Height in ASD vs Controls | | |  |
| --- | --- | --- | --- | --- | --- | --- | --- | --- | --- |
| Age (months) | ASD / Controls  N | ASD Mean (*SD*) | Controls Mean (*SD*) | T-Test | FDR adjusted p value | ASD Mean (*SD*) | Controls Mean (*SD*) | T-Test | FDR adjusted p value |
| 0 | 61 / 4,948 | 35.18 (1.23) | 34.92 (1.37) | T (5,007) = -1.48, p = 0.14 | 0.21 | 51.03 (2.55) | 50.89 (2.38) | T (5007) = - 0.46, p = 0.65 | 0.73 |
| 1 | 48 / 4,215 | 39.10 (1.41) | 38.76 (1.32) | T (4,261) = -1.75, p = 0.080 | 0.14 | 57.69 (2.89) | 57.19 (2.51) | T (4,261) = - 1.36, p = 0.17 | 0.31 |
| 2 | 23 / 1,751 | 40.40 (1.08) | 39.60 (1.43) | **T (1,772) = -2.69, p = 0.007** | 0.063 | 59.83 (2.52) | 58.83 (2.91) | T (1,772) = -1.63, p = 0.10 | 0.23 |
| 3 | 10 / 875 | 42.03 (1.97) | 41.16 (1.38) | **T (883) = -1.97, p = 0.049** | 0.11 | 63.45 (3.86) | 62.06 (2.54) | T (883) = -1.71, p = 0.09 | 0.23 |
| 4 | 11 / 741 | 42.06 (1.50) | 41.71 (1.44) | T (750) = -0.82, p = 0.42 | 0.54 | 62.35 (5.18) | 62.96 (2.65) | T (750) = 0.74, p = 0.46 | 0.69 |
| 8 | 20 / 1,953 | 45.32 (1.51) | 45.35 (1.48) | T (1,971) = 0.10, p = 0.92 | 0.97 | 71.10 (2.90) | 71.09 (2.76) | T (1,971) = -0.02, p = 0.98 | 0.98 |
| 9 | 42 / 3,075 | 46.36 (1.54) | 45.85 (1.40) | **T (3,115) = -2.35, p = 0.019** | 0.089 | 73.37 (3.21) | 72.28 (2.87) | **T (3,115) = -2.43, p = 0.015** | 0.068 |
| 12 | 17 / 934 | 46.61 (1.79) | 46.64 (1.44) | T (949) = 0.04, p = 0.97 | 0.97 | 76.07 (2.91) | 75.74 (2.59) | T (949) = -0.53, p = 0.60 | 0.73 |
| 186 (15.5 years) | 33 / 3,188 | 56.12 (1.82) | 55.50 (1.73) | **T (3,219) = -2.02, p = 0.043** | 0.97 | 172.91 (7.85) | 169.34 (8.24) | **T (3,219) = -2.47, p = 0.013** | 0.068 |

### Sensitivity Analyses (ASD)

**Table S2.** T-tests of head circumference values from birth to 12 months in ASD and controls. Head circumference at age 15.5 years is also reported.

To enable more comparability across groups by basing analyses from the same analytic sample, within our sensitivity analyses we only included participants with complete data on both outcome variables (ASD and autistic traits). A total of 5,636 had complete data on both clinical measures and confounders. From this sample, there were a total of 69 ASD cases and 487 participants in the autistic traits group.

In our sensitivity analyses we restricted the sample to those with complete data on both clinical outcomes and confounders. Across participants, the mean number of timepoints at which head circumference data was available was 5 (SD = 3.28, range: 1 - 18). 96.90% of participants were of white ethnicity, with 3.10% of non-white ethnicity. Within ASD cases, 100% of participants were of white ethnicity. In the high autistic traits group, 3.34% were of an ethnic minority. Length and weight at birth was on average larger in ASD, although only height at age 15.5 years was significantly larger in the ASD group compared to controls (t(2907) = -2.65, p = 0.008).

ASD sensitivity analyses showed similar results to the main analyses, i.e., when participants had complete data for both clinical outcomes (ASD and autistic traits), those with a diagnosis of ASD had higher head circumference values than controls (see Table S3).

**Table S5.** Output from ASD sensitivity analyses (1). Univariable and multivariable linear mixed regressions assessing head circumference in autism versus controls. Sample based on timepoints with at least 10 participants per group, and complete data for all clinical measures (ASD and autistic traits) and confounders. (N = 5,547, ASD = 68, Controls = 5,479)

| Sensitivity: Head circumference trajectories in **ASD vs Controls** | | | |
| --- | --- | --- | --- |
|  | Beta Coefficient | 95% Confidence intervals | *P* |
| Univariable model 1 | 0.78 | 0.36 – 1.20 | <0.0001 |
| Model 2: model 1 + child’s sex | 0.50 | 0.09 – 0.90 | 0.016 |
| Model 3: model 2 + maternal education | 0.50 | 0.09 – 0.90 | 0.017 |
| Model 4: model 3 + maternal BMI | 0.50 | 0.10 – 0.90 | 0.015 |
| Model 5: model 4 + gestational age and birth weight | 0.45 | 0.06 – 0.84 | 0.024 |
| Model 6: model 5 + height | 0.05 | -0.24 – 0.33 | 0.75 |

Univariable model 1 consisted of ASD and time variables. Interactions between ASD and age (p = 0.93), age squared (p = 0.32), and sex (p = 0.59) were non-significant and therefore the group coefficient was not included in this table.

### Head circumference and height across first 12 months in autistic traits group: T-tests

Head circumference did not significantly differ between elevated autistic traits and controls at birth through to 5 months (**Table S6**), although head circumference was significantly smaller in the high autistic traits group compared to controls at 6 months (t[269] = 2.01, p = 0.045) and 7 months (t[319] = 3.23, p = 0.001). No other significant differences in head circumference size were observed in the first year of life or at age 15 years. Height was significantly smaller in the autistic traits group relative to controls at age 3 (t[942] = 2.15, p = 0.032) and 7 months (t[319] = 2.42, p = 0.016).

**Table S6.** T-tests of head circumference values from birth to 12 months in Autistic traits and controls. Head circumference at age 15.5 years is also reported.

|  | | Head Circumference in Autistic Traits vs Controls | | | | Height in Autistic Traits vs Controls | | | |
| --- | --- | --- | --- | --- | --- | --- | --- | --- | --- |
| Age (months) | Autistic Traits / Controls  N | Autistic Traits Mean (*SD*) | Controls Mean (*SD*) | T-Test | FDR adjusted p value | Autistic Traits Mean (*SD*) | Controls Mean (*SD*) | T-Test | FDR adjusted p value |
| 0 | 504 / 4,771 | 34.83 (1.45) | 34.92 (1.37) | T (5,243) = 1.50, p = 0.13 | 0.30 | 50.70 (2.54) | 50.90 (2.37) | T (5,273) = 1.73, p = 0.083 | 0.39 |
| 1 | 403 / 4,070 | 38.71 (1.34) | 38.78 (1.31) | T (4,471) = 0.95, p = 0.34 | 0.43 | 57.24 (2.85) | 57.20 (2.45) | T (4,471) = -0.29, p = 0.78 | 0.78 |
| 2 | 195 / 1,686 | 39.63 (1.61) | 39.61 (1.40) | T (1,879) = -0.20, p = 0.84 | 0.84 | 58.76 (3.26) | 58.85 (2.90) | T (1,879) = 0.40, p = 0.69 | 0.74 |
| 3 | 83 / 861 | 40.89 (1.63) | 41.19 (1.38) | T (942) = 1.90, p = 0.058 | 0.23 | 61.46 (3.08) | 62.12 (2.63) | **T (942) = 2.15, p = 0.032** | 0.22 |
| 4 | 81 / 720 | 41.58 (1.68) | 41.71 (1.44) | T (799) = 0.71, p = 0.48 | 0.51 | 62.81 (3.11) | 62.95 (2.69) | T (799) = 0.46, p = 0.64 | 0.74 |
| 5 | 31 / 240 | 43.07 (1.56) | 43.55 (1.46) | T (291) = 1.70, p = 0.09 | 0.25 | 66.48 (4.02) | 67.12 (3.43) | T (291) = 0.96, p = 0.34 | 0.72 |
| 6 | 31 / 240 | 43.79 (269) | 44.33 (1.40) | **T (269) = 2.01, p = 0.045** | 0.23 | 67.97 (4.03) | 68.42 (3.23) | T (269) = 0.72, p = 0.47 | 0.73 |
| 7 | 41 / 280 | 44.01 (1.87) | 44.85 (1.50) | **T (319) = 3.23, p = 0.001** | **0.014** | 68.97 (3.54) | 70.15 (2.80) | **T (319) = 2.43, p = 0.016** | 0.23 |
| 8 | 193 / 1,859 | 45.29 (1.65) | 45.37 (1.47) | T (2,050) = 0.72, p = 0.47 | 0.51 | 70.91 (3.12) | 71.10 (2.78) | T (2,050) = 0.91, p = 0.36 | 0.72 |
|  |  |  |  |  |  |  |  |  |  |
| 9 | 317 / 3,012 | 45.78 (1.53) | 45.86 (1.39) | T (3,327) = 1.05, p = 0.29 | 0.43 | 72.20 (2.98) | 72.37 (2.91) | T (3,327) = 1.00, p = 0.32 | 0.72 |
| 10 | 70 / 605 | 46.51 (1.49) | 46.31 (1.51) | T (673) = -1.08, p = 0.28 | 0.43 | 74.11 (3.32) | 73.50 (3.05) | T (673) = -1.57, p = 0.12 | 0.42 |
| 11 | 35 / 300 | 46.40 (1.67) | 46.86 (1.53) | T (333) = -1.68, p = 0.09 | 0.25 | 74.67 (3.78) | 75.09 (3.12) | T (333) = 0.75, p = 0.46 | 0.73 |
| 12 | 97 / 918 | 46.47 (1.81) | 46.66 (1.46) | T (1,013) = 1.20, p = 0.23 | 0.43 | 75.82 (2.99) | 75.73 (2.70) | T (3,239) = -0.57, p = 0.57 | 0.73 |
| 186 (15.5 years) | 261 / 2,980 | 55.41 (1.88) | 55.52 (1.71) | T (3,239) = 1.02, p = 0.31 | 0.43 | 169.69 (8.94) | 169.37 (8.33) | T (3,239) = -0.57, p = 0.57 | 0.73 |

### Sensitivity analyses (autistic traits)

Reduced head circumference was observed in the autistic traits group (see Table S7). When we removed those with a diagnosis of ASD from trait analyses, more marked reductions in head circumference were observed see (Table S8).

**Table S7.** Output from autistic traits sensitivity analyses (1). Univariable and multivariable linear mixed regressions assessing head circumference in autistic traits versus controls. Sample based on timepoints with at least 10 participants per group, and complete data for all clinical measures (ASD and autistic traits) and confounders. (N = 5,547, autistic traits = 478, controls = 5,069)

| Sensitivity: Head circumference trajectories in **Autistic Traits Group vs Controls** | | | |
| --- | --- | --- | --- |
|  | Beta Coefficient | 95% Confidence intervals | *P* |
| Univariable model 1 | -0.05 | -0.22 – 0.11 | 0.55 |
| Model 2: model 1 + child’s sex | -0.18 | -0.34 - -0.02 | 0.026 |
| Model 3: model 2 + maternal education | -0.17 | -0.32 - -0.01 | 0.041 |
| Model 4: model 3 + maternal BMI | -0.16 | -0.32 - -0.005 | 0.043 |
| Model 5: model 4 + gestational age and birth weight | -0.12 | -0.27 – 0.03 | 0.12 |
| Model 6: model 5 + height | -0.06 | -0.17 – 0.05 | 0.29 |

Univariable model 1 consisted of autistic traits and time variables. Interactions between ASD and age (p = 0.63), age squared (p = 0.68) ,and sex (p = 0.09) were non-significant and therefore the group coefficient was not included in this table.

**Table S8.** Output from autistic traits sensitivity analyses (2). Univariable and multivariable linear mixed regressions assessing head circumference in autistic traits versus controls. We removed individuals with ASD so that we could assess mean differences in head circumference in a subclinical sample. Sample based on timepoints with at least 10 participants per group, and complete data for all clinical measures (ASD and autistic traits) and confounders. (N = 5,479; Autistic traits = 434, Controls = 5,045).

| Sensitivity: Head circumference trajectories in **Autistic Traits Group vs Controls** | | | |
| --- | --- | --- | --- |
|  | Beta Coefficient | 95% Confidence intervals | *P* |
| Univariable model 1 | -0.15 | -0.32 – 0.03 | 0.096 |
| Model 2: model 1 + child’s sex | -0.25 | -0.42 - -0.09 | 0.003 |
| Model 3: model 2 + maternal education | -0.24 | -0.40 - -0.07 | 0.005 |
| Model 4: model 3 + maternal BMI | -0.24 | -0.40 - -0.07 | 0.005 |
| Model 5: model 4 + gestational age and birth weight | -0.19 | -0.35 - -0.03 | 0.017 |
| Model 6: model 5 + height | -0.09 | -0.20 - -0.03 | 0.15 |

For univariable models 2 onwards, we incorporated random effects of participant id and mean centred age. Univariable model 1 consisted of autistic traits and time variables. Interactions between ASD and age (p = 0.66), age squared (p = 0.83), and sex (p = 0.15) were non-significant and therefore the group coefficient was not included in this table.

## Sample flowcharts

Figure S2: Flowchart of study participation (ASD analyses)

**N = 15,645 (100%)**

**Full ALSPAC sample**

N = 15,442

Singleton birth / one twin

N = 14,197

At least one head circumference measurement

N = 1,245

No head circumference measurement

N = 10,604

Complete covariate / confounder data

N = 3,593

Missing data on height, weight at birth, sex, maternal education, maternal BMI, gestational age.

N = 6,492

Completed autism outcome measure

N = 3,112

No data on autism diagnosis

**N = 6,482 (41.49%)**

**Final sample**

N = 10

Outliers / Biologically implausible values for head circumference, height, weight at birth. Less than 10 participants per timepoint in each group.

Figure S3: Flowchart of study participation (autistic traits analyses)

**N = 15,645 (100%)**

**Full ALSPAC sample**

N = 14,442

Singleton birth / one twin

N = 14,197

At least one head circumference measurement

N = 1,245

No head circumference measurement

N = 10,604

Complete covariate / confounder data

N = 3,593

Missing data on height, weight at birth, sex, maternal education, maternal BMI, gestational age.

N = 6,872

Autistic traits outcome data (SCDC)

N = 3,732

No data on autistic traits

**N = 6,869 (43.92%)**

**Final sample**

N = 3

Outliers / Biologically implausible values for head circumference, height, weight at birth. Less than 10 participants per timepoint in each group.

Figure S4: Flowchart of study participation (complete data on both clinical outcomes: autism and autistic traits)

**N = 15,645 (100%)**

**Full ALSPAC sample**

N = 14,442

Singleton birth / one twin

N = 14,197

At least one head circumference measurement

N = 1,245

No head circumference measurement

N = 10,604

Complete covariate / confounder data

N = 3,593

Missing data on height, weight at birth, sex, maternal education, maternal BMI, gestational age.

N = 5,642

Complete data on both clinical outcomes (autism and autistic traits)

N = 4,962

No data on autism and/or autistic traits

**N = 5,636 (36.04%)**

**Final sample**

N = 8

Outliers / Biologically implausible values for head circumference, height, weight at birth. Less than 10 participants per timepoint in each group.
